# Structural Abnormalities in Childhood Absence Epilepsy: Voxel-Based Analysis Using Diffusion Tensor Imaging

**DOI:** 10.3389/fnhum.2016.00483

**Published:** 2016-09-28

**Authors:** Wenchao Qiu, Yuan Gao, Chuanyong Yu, Ailiang Miao, Lu Tang, Shuyang Huang, Zheng Hu, Jing Xiang, Xiaoshan Wang

**Affiliations:** ^1^Department of Neurology, Nanjing Brain Hospital, Nanjing Medical UniversityNanjing, China; ^2^Department of Neurology, Nanjing Children’s HospitalNanjing, China; ^3^MEG Center, Division of Neurology, Cincinnati Children’s Hospital Medical CenterCincinnati, OH, USA

**Keywords:** childhood absence epilepsy, diffusion tensor imaging, voxel-based analysis, default mode network, structural impairment

## Abstract

**Purpose:** Childhood absence epilepsy (CAE) is a common syndrome of idiopathic generalized epilepsy. However, little is known about the brain structural changes in this type of epilepsy, especially in the default mode network (DMN) regions. This study aims at using the diffusion tensor imaging (DTI) technique to quantify structural abnormalities of DMN nodes in CAE patients.

**Method:** DTI data were acquired in 14 CAE patients (aged 8.64 ± 2.59 years, seven females and seven males) and 16 age- and sex-matched healthy controls. The data were analyzed using voxel-based analysis (VBA) and statistically compared between patients and controls. Pearson correlation was explored between altered DTI metrics and clinical parameters. The difference of brain volumes between patients and controls were also tested using unpaired *t*-test.

**Results:** Patients showed significant increase of mean diffusivity (MD) and radial diffusivity (RD) in left medial prefrontal cortex (MPFC), and decrease of fractional anisotropy (FA) in left precuneus and axial diffusivity (AD) in both left MPFC and precuneus. In correlation analysis, MD value from left MPFC was positively associated with duration of epilepsy. Neither the disease duration nor the seizure frequency showed significant correlation with FA values. Between-group comparison of brain volumes got no significant difference.

**Conclusion:** The findings indicate that structural impairments exist in DMN regions in children suffering from absence epilepsy and MD values positively correlate with epilepsy duration. This may contribute to understanding the pathological mechanisms of chronic neurological deficits and promote the development of new therapies for this disorder.

## Introduction

Childhood absence epilepsy (CAE) is an idiopathic, generalized and non-convulsive event characterized by frequent and brief loss of consciousness, as well as bilateral and synchronous discharges of 3 Hz generalized spike-and-wave (SWDs) recorded on electroencephalography (Engel and International League Against Epilepsy (ILAE), 2001). Accounting for 10–17% of all cases of epilepsy diagnosed in school-aged children, the CAE generally onset between the age of 3 and 8 years (peak in 6–7 years; Tenney and Glauser, 2013). Although CAE seems clinically benign, a growing interest in the cognitive and behavioral performances of these patients have been aroused, and significant neuropsychological deficits were also uncovered which suggested a further effect of absence of seizures on brain’s function (Caplan et al., 2008; Masur et al., 2013).

In past decades, an increasing number of neuroimaging studies have focused on this kind of patients and indeed got some important achievements. Higher fMRI signal was observed in several cortical and subcortical areas, such as the thalamus, somatosensory and motor cortex, during ictal SWDs (Berman et al., 2010) and interictal SWDs (Li et al., 2009) in CAE patients. Recent fMRI studies found that functional connectivity between different regions also altered even when there were no SWDs (Killory et al., 2011; Masterton et al., 2012), which indicated that brain function of CAE patients may be disrupted all the time even during resting state without epileptic seizure. These findings were further supported by some diffusion tensor imaging (DTI) studies. For example, as structural basis of functional connectivity between remote brain regions, impaired white matter integrity of fibers connecting thalamus and cortex were revealed in a previous study (Yang et al., 2012). Among these fMRI and EEG-fMRI studies concerning CAE, abnormal functional connectivity in default mode network (DMN) was also detected (Luo et al., 2011). The DMN is a baseline state brain network, consisting of medial prefrontal cortex (MPFC), posterior cingulate cortex/precuneus (PCC), bilateral inferior parietal cortex and bilateral inferior temporal cortex (Raichle et al., 2001). Negatively associated with goal-directed cognitive task, DMN is responsible for person’s conscious state, and moreover, may function as a biomarker of consciousness disability (Crone et al., 2011). However, the structural neuroimaging changes in DMN nodes have not been previously determined. Given that classical feature of CAE is the transient conscious disruption and CAE was found to be associated with epileptic discharges in DMN nodes in our previous magnetoencephalography (MEG) study (Miao et al., 2014), we mainly focus on whether structural damage exists in DMN regions of CAE patients using DTI. Although DTI has been used to study structural changes in CAE (Xue et al., 2014), they did a whole brain structural connectivity analysis instead of focusing on DMN and the deterministic tractography by fiber assignment by continuous tracking (FACT) algorithm employed in their study is under heavy debate when it comes to the fiber-crossing problem within per-voxel. In this article, we took a voxel-based analysis (VBA) to determine whether DTI parameters in DMN nodes would change in CAE patients compared with those age- and sex-matched healthy controls.

## Materials and Methods

### Participants

Fourteen children with absence epilepsy from the Neurology Department of Nanjing Children’s Hospital were recruited, including seven males and seven females aged 6–13 years (mean ± SD 8.64 ± 2.59 years). All of them attended regular schools. The diagnoses of typical absence epilepsy were established according to the recommendations of the International League Against Epilepsy (ILAE; Engel and International League Against Epilepsy (ILAE) (2001)). The inclusion criteria were: (1) clinical EEG recordings with bilateral, synchronous, symmetrical discharges of approximate 3 Hz spike waves on normal background; (2) normal neurological and general physical examination; and (3) no abnormalities were found during routine structural MRI scanning. Patients with mental disorders or cognitive impairments were excluded. Sixteen age- and gender-matched healthy controls aged 6–13 years (mean ± SD 9.13 ± 2.95, nine males and seven females), who had no history of neurological or psychiatric disorders and no structural abnormalities during a routine MRI examination, were recruited. There were no significant differences in age (*p* = 0.69, *t*-test) or gender (*p* = 0.73, chi-square test) between the two groups. All participants in our study were right-handed.

At the time of participating in this study, six patients had no antiepileptic drug, eight had one antiepileptic drug and no one had two or more drugs (see Table 1). Written informed consent to participate in the study was obtained from their guardians. Our study was reviewed and approved by the ethical boards of Nanjing Children’s Hospital, Nanjing Brain Hospital, and Nanjing Medical University.

**Table 1:** **Clinical information for childhood absence epilepsy (CAE) patients**.

Subjects ID	Sex	Age (y)	Disease duration (m)	Frequency of seizure (times/d)	AED treatment	Frequency of SWDs (Hz)
1	M	7	7	7–8	VAL	2–3
2	M	6	12	3–4	VAL	2.5–3
3	M	12	7	10	None	3
4	F	12	3	10–15	None	2.5–3.5
5	F	11	9	6–7	LEV	3
6	M	8	19	1–2	VAL	3–3.5
7	F	7	13	10–15	VAL	3
8	F	6	14	3–4	LEV	2–3
9	M	8	5	6–7	None	3
10	F	6	20	1–2	None	3
11	F	6	4	5–6	None	3
12	M	13	18	2–3	VAL	3
13	F	8	4	15–20	None	3–3.2
14	M	11	11	5–6	VAL	3

*M, male; F, female; d, day; m, months; y, years; AED, antiepileptic drug; LEV, levetiracetam; VAL, sodium valproate*.

### Image Acquisition

Magnetic resonance images were acquired using a 3.0T MRI scanner (Siemens, Germany) with an 8-channel phased array head coil in all patients and controls. All subjects were instructed to minimize their head movements during the scanning. First, anatomic 3D T1-weighted images were obtained using a rapid gradient echo sequence (TR/TE = 1900/2.48 ms). The imaging parameters were as follows: field of view was 250 × 250 mm; flip angle was 9°; matrix = 512 × 512. For each subject, 176 sagittal slices were collected. Then, the diffusion images were collected with scanning parameters as follows: a single shot echo planar imaging sequence (TR/TE = 6600/93 ms) was employed; the number of average was 1; slice thickness = 3 mm with no gap; field of view = 240 × 240 mm; matrix = 128 × 128. One non-diffusion weighted volume (*b* = 0 s/mm^2^) and 30 diffusion-weighted volumes (*b* = 1000 s/mm^2^) encoded in 30 non-collinear gradient directions were acquired. For each volume, 45 axial slices were collected.

### Image Processing

The eddy current distortions and head motion in original DTI data were corrected by affine co-registration to B0 image using FSL’s (FMRIB Software Library^1^) “eddy current correction” (Jenkinson and Smith, 2001).

Then, in subject’s native space, fractional anisotropy (FA), mean diffusivity (MD), radial diffusivity (RD) and axial diffusivity (AD) maps of all participants were calculated in each voxel using DTIFIT^2^. To bring the resulting maps from native space into standard Montreal Neurological Institute (MNI) space, all of the B0 images were normalized to MNI space to estimating the normalization parameters using SPM12 (Statistical Parametric Mapping^3^), running in MATLAB R2012b (Math Works, Natick, MA, USA). Using the EPI template supplied by SPM12, original voxel size of 1.875 × 1.875 × 3 mm was interpolated to a standard voxel size of 2 × 2 × 2 mm and estimated parameters were written to the corresponding diffusion maps (FA, MD, RD and AD maps). Finally, the normalized maps were spatially smoothed with a 4 mm full-width at half-maximum (FWHM) kernel to improve the signal-to-noise ratio (SNR).

To search for between-group differences in brain volume, we used an optimized procedure provided by VBM8 toolbox^4^ on the T1 images. First, all T1 images were bias corrected, optimally normalized using rigid-body transformation and segmented using the “unified segmentation” process (Ashburner and Friston, 2000). Then, the gray and white matter images were spatially smoothed with an 8 mm FWHM kernel. Finally, volumes of gray matter, white matter and cerebral spinal fluid were automatically calculated through VBM8.

### Statistical Analysis

An unpaired two-sample *t*-test was performed by contrasting DTI parametric maps to examine the difference between CAE patients and healthy controls. The general linear model, implemented in SPM12, was used for statistical analysis. Both age and gender were taken as covariates of no interest, for that these measures might influence the brain structure development (Snook et al., 2005). We employed the false discovery rate (FDR) to correct for multiple comparisons (Genovese et al., 2002), and a *p*-value < 0.05 after correction was considered as statistically significant.

SPSS v19.0 was taken as statistical analysis software for brain volume comparison. The difference of brain volumes (e.g., total brain volume, gray matter volume and white matter volume) between patients and controls were tested using unpaired *t*-test (*p* < 0.05). Moreover, the relative gray matter volume and white matter volume was also analyzed in our study. This was achieved by dividing gray matter and white matter volume by whole brain volume and expressed as a percentage score.

Pearson correlation was performed to examine the relationship between FA, MD values and frequency of seizures and epilepsy duration in CAE group. Given the relatively short duration of illness in these subjects, we also examined the association with age of onset. For FA and MD values, clusters with significant between-group difference detected in our group analysis of DTI maps were taken as ROI and the mean FA and MD values in these regions were extracted.

## Results

**Figure 1:**
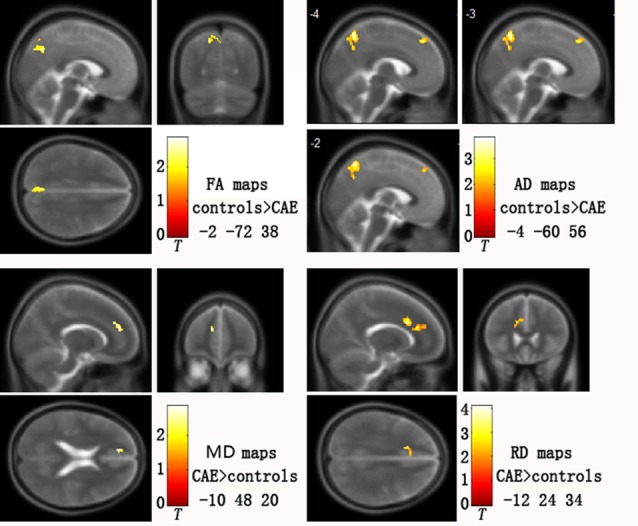
**Abnormal diffusion tensor imaging (DTI) parameters in childhood absence epilepsy (CAE) patients compared with healthy controls.** Clusters are superimposed on the T2 templates in XJVIEW for fractional anisotropy (FA), mean diffusivity (MD), axial diffusivity (AD) and radial diffusivity (RD). Coordinates (*x, y, z*) show the slice on the sagittal top left (*x*), coronal top right (*y*) and horizontal bottom left (*z*) view of each template set. For AD maps, three sagittal slices are shown to better display the two clusters.

Group analysis showed statistically significant differences in DTI parametric images between patients and healthy controls after correction for multiple comparisons. Specifically, patients showed: (i) increased MD in left medial prefrontal gyrus (*p* = 0.042); (ii) increased RD in left medial prefrontal gyrus (*p* = 0.006); (iii) decreased FA in left precuneus (*p* = 0.010); and (iv) decreased AD in both the left medial prefrontal gyrus and precuneus (*p* = 0.000; for details, see Figure 1 and Table 2). There was no increased FA or AD in patients compared with controls, and no decreased MD or RD.

**Table 2:** **Areas of significant change in DTI metrics**.

DTI parameters	AAL	*k*	Peak MNI (*x,y,z*)			*p*-value
MD elevation	Frontal_Sup_Medial_L	101	−12	48	20	0.042
RD elevation	Frontal_Sup_Medial_L	211	−14	24	34	0.006
FA reduction	Precuneus_L	176	−2	−72	38	0.010
AD reduction	Precuneus_L	396	−4	−60	56	0.000
	Frontal_Sup_Medial_L	126				

*DTI, diffusion tensor imaging; FA, fractional anisotropy; MD, mean diffusivity; RD, radial diffusivity; AD, axial diffusivity; AAL, automated anatomical labeling atlas; MNI, Montreal Neurological Institute*.

**Figure 2:**
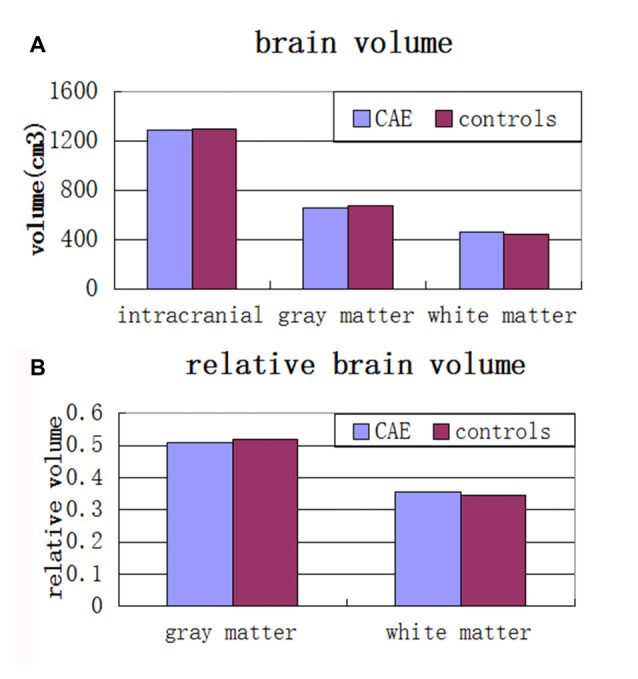
**(A)** Mean total intracranial, gray and white matter volumes and **(B)** relative gray matter and white matter volumes in children with absence epilepsy compared to healthy controls. There were no significant group differences across any of these volumes.

There were no significant differences between the CAE and control groups in brain volumes. Specifically, there were no significant differences for total brain volume (*p* = 0.93), gray matter volume (*p* = 0.46), white matter volume (*p* = 0.63), relative gray matter volume (*p* = 0.10) or relative white matter volume (*p* = 0.29; see Figure 2).

**Figure 3:**
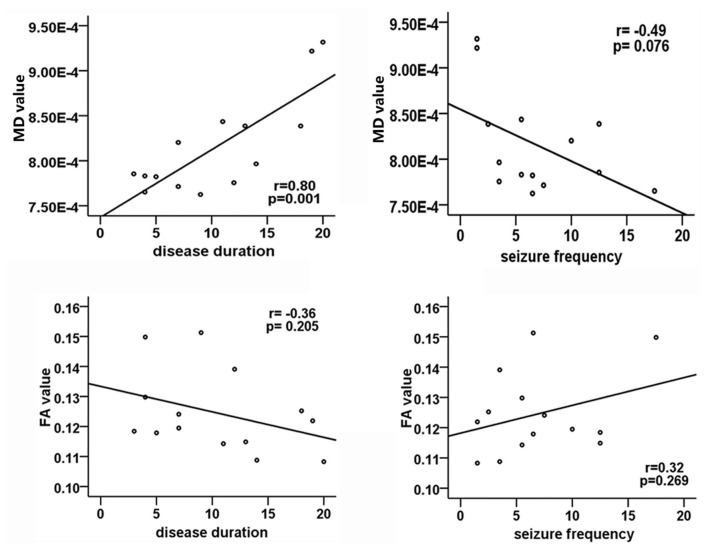
**Correlation analysis between DTI metrics and clinical parameters in patients group.** The Pearson correlation coefficient and *p* value are plotted in each case. Significant correlation only exists between MD values and disease duration.

Positive correlation was found between MD values and the duration of epilepsy(*r* = 0.80, *p* = 0.001; Figure 3). No significant relationship between FA values and disease duration or seizure frequency was detected, and the age of onset in patients was not associated with DTI metrics.

## Discussion

In this study, we used DTI and VBA to explore the brain microstructural abnormalities of DMN nodes in CAE patients. Significant changes of DTI metrics were uncovered in these regions in CAE patients compared with control subjects. Moreover, the data successfully support the existence of correlation between the duration of disease and MD values. Although our study aimed at structural abnormalities in DMN regions, we rejected the ROI based analysis because of the significant inter-subject variability in manually delineating ROIs. Instead, we employed a fully automated brain measurement technique, VBA, to register all subjects’ brain images to the MNI template.

DTI is sensitive to the diffusion patterns of water molecules based on an ellipsoid model. Combination of FA, MD, RD and AD has already been proven of great importance in exploring different mechanisms underlying the microstructural changes (Di Paola et al., 2010). In epilepsy, DTI has also showed growing use in human patients (Rugg-Gunn et al., 2001, 2002; Grant, 2005; Luat and Chugani, 2008) by identifying potentially epileptogenic lesions, providing information about pathways for seizure propagation, and detecting abnormalities even when conventional MRI is normal. In this study, DTI impairments were found in several regions, which may result from epileptic seizures. The decreased FA and AD in left precuneus might reflect a tissue containing less number of similarly aligned neurons (Mukherjee et al., 2008), which indicated a lower cellular density with less cytomembrane barriers resulting from chronic epileptic discharges. In addition, altered FA might also mirror a structural disorganization of the tissue and changes in myelinated axonal density (Takahashi et al., 2002; Schulte et al., 2005). However, there is no histopathologic evidence supporting this hypothesis directly, which needs to be further explored in future. The possible explanation for elevated MD and RD in left medial prefrontal gyrus might be the expansion of extracellular space due to chronic effects of epileptic discharge. Previous studies have confirmed that the excitotoxic mechanisms caused by seizures may lead to cell lysis and death (Wasterlain et al., 1993; Yogarajah and Duncan, 2008), which may result in an increase in extracellular space and then give rise to an increased diffusion. Of noteworthy, MD is not a much stable measure and could be highly affected by external factors such as antiepileptic medication, cortical edema and some other reasons.

In the current study, aberrant DTI indices are mainly located in DMN nodes including left medial prefrontal gyrus and precuneus. These findings are consistent with previous resting-state fMRI study (Yang et al., 2014), which revealed decreased ReHo in precuneus and several other regions. Besides the DMN, many other networks involved in CAE were detected by fMRI, such as salience network, cognitive control network and affective network (Yang et al., 2013; Luo et al., 2014). However, DMN was identified as a core network with changed activity central to absence seizure and interictal epileptiform discharges (Carney and Jackson, 2014), and our previous MEG study also support the involvement of DMN in initialization of epileptiform discharges in CAE (Miao et al., 2014). Moreover, altered functional connectivity strength of DMN also exists in CAE patients compared with healthy controls (Li et al., 2015). All these studies indicate that abnormal DTI parameters found in our study may be associated with the long-term dysfunctions in DMN. However, whether structural abnormalities are the result or reason of dysfunctions is still unclear up to now. Considering that children with new-onset epilepsy had already exhibited diffusion abnormalities (Hutchinson et al., 2010; Widjaja et al., 2013), we tentatively hold the point that structural abnormalities may be the reason of dysfunction, which needs to be further proved in the future. Given that DMN is highly associated with cognitive impairment in some mental disorders (Buckner et al., 2008) and CAE patients also suffer from some cognitive and memory problems (Nolan et al., 2004), we can make a heavy hypothesis that cognitive dysfunctions reported in CAE may somehow correlated with structural abnormalities of DMN nodes in this type of epilepsy. This may be of great importance in evaluating patients’ illness state and prognosis. Unfortunately, because of some practical limitations, present study did not assess the cognitive performance of patients, but we will strengthen our work by some cognitive questionnaires in the future.

In the between-group comparison of brain volume, no significant difference was detected between CAE patients and control subjects in our study. However, another brain volume study revealed that CAE patients got smaller gray matter volumes in both frontal and temporal lobes (Caplan et al., 2009) and Hermann et al. (2010) also went to a conclusion that children with epilepsy exhibited an aberrant pattern of white matter volume development in a prospective study. The discrepancy between our study and the previous ones may be explained by the medication usage, small sample size and some other factors (e.g., data themselves) in our research.

Many studies have reported significant correlation between the DTI metrics and clinical symptoms in patients with epilepsy (Keller et al., 2011, 2012). In the current research, significant positive correlation was also found between MD values and duration of epilepsy, which means that longer the duration, the worse damage occur in the brain of CAE. Given that DMN is associated with cognitive function as discussed above, more damage in DMN nodes may lead to a worse cognitive performance and then a lower academic achievement during school life, which seems terrible to childhood patients. As a result, the reasonable and positive therapies seem of vital importance to protect this kind of patient from epileptiform discharges and structural impairment as soon as possible.

Finally, we acknowledge that the present study has several limitations. First, group size in our study was small and the findings of this study should be considered as preliminary which needs to be confirmed and extended with more subjects, including both patients and healthy volunteers. Second, the antiepileptic drugs taken by eight of our patients might lead to the alteration of brain metabolism, and the effects of medications would be considered in the future. Third, VBA was used instead of manually delineating ROIs considering the inter-subject variability. But normalizing the results of children to standard MNI space may involve a higher degree of non-linear transformations and associated misregistration issues, which may then give rise to some false positive clusters. However, as mentioned in a previous study (Rugg-Gunn et al., 2001), we tried to limit the possibility of obtaining a false positive cluster by using a relatively strict restriction of *P* < 0.05 (FDR corrected).

In conclusion, the present study found structural abnormalities of DMN regions in CAE patients by DTI. Moreover, pearson correlation analysis showed that MD values from left MPFC positively correlated with duration of epilepsy. Aberrant DTI metrics of DMN regions in CAE patients provide a structural evidence to explain the functional abnormalities detected in fMRI studies and may become a new biomarker for CAE diagnosis. We hope our study contributes to understanding the pathological mechanisms of chronic neurological deficits in children suffering from absence epilepsy, and may help the explorations of new treatment for this disorder.

## Author Contributions

WQ, YG and XW designed the study. YG, CY, ZH and AM acquired the data, while WQ, LT and SH analyzed the data together. WQ and YG wrote the manuscript, while JX and XW revised it. All authors signed the final approval for publication.

## Conflict of Interest Statement

The authors declare that the research was conducted in the absence of any commercial or financial relationships that could be construed as a potential conflict of interest.

## Footnotes

^1^http://www.fmrib.ox.ac.uk/fsl/

^2^http://www.fmrib.ox.ac.uk/fsl/fdt/fdt_dtifit.html

^3^http://www.fil.ion.ucl.ac.uk/spm/software/spm12/

^4^http://dbm.neuro.uni-jena.de/vbm.html
